# Editorial: Cellular and molecular mechanisms of lung regeneration, repair, and fibrosis

**DOI:** 10.3389/fcell.2023.1346875

**Published:** 2024-01-08

**Authors:** Chunheng Mo, Mengli Yan, Xiao Xiao Tang, Shigeyuki Shichino, Gianluca Bagnato

**Affiliations:** ^1^ Key Laboratory of Birth Defects and Related Diseases of Women and Children of MOE, State Key Laboratory of Biotherapy, West China Second University Hospital, Sichuan University, Chengdu, China; ^2^ Institute of Hematology, Henan Key Laboratory of Stem Cell Differentiation and Modification, Henan Provincial People’s Hospital, Zhengzhou University People’s Hospital, Henan University People’s Hospital, Zhengzhou, Henan, China; ^3^ State Key Laboratory of Respiratory Disease, National Clinical Research Center for Respiratory Disease, National Center for Respiratory Medicine, Guangzhou Institute of Respiratory Health, The First Affiliated Hospital of Guangzhou Medical University, Guangzhou, China; ^4^ Guangzhou Laboratory, Bio-Island, Guangzhou, China; ^5^ Division of Molecular Regulation of Inflammatory and Immune Diseases, Research Institute of Biomedical Sciences, Tokyo University of Science, Chiba, Japan; ^6^ Department of Clinical and Experimental Medicine, University of Messina, Messina, Italy

**Keywords:** lung fibrosis, lung regeneration, lung repair, cellular and molecular mechanisms, machine learning

Organ fibrosis poses a significant threat to human health, contributing to over 30% of diseases that result in disability and mortality (Rockey et al., 2015; Zhou et al., 2022; Bhattacharya and Ramachandran, 2023; Yan et al., 2023). Idiopathic pulmonary fibrosis (IPF) is a progressive and often fatal lung disease characterized by fibrosis. Unfortunately, the median survival for IPF patients is only 3–5 years. Currently, the FDA-approved oral agents for pulmonary fibrosis, Pirfenidone and Nintedanib, can only slow down disease progression and do not provide a cure. Therefore, there is an urgent need to develop more effective therapeutic approaches for lung fibrosis and gain a deeper understanding of the cellular and molecular mechanisms underlying this condition (Zhang et al., 2023a; Zhang et al.; Podolanczuk et al., 2023).

**FIGURE 1:**
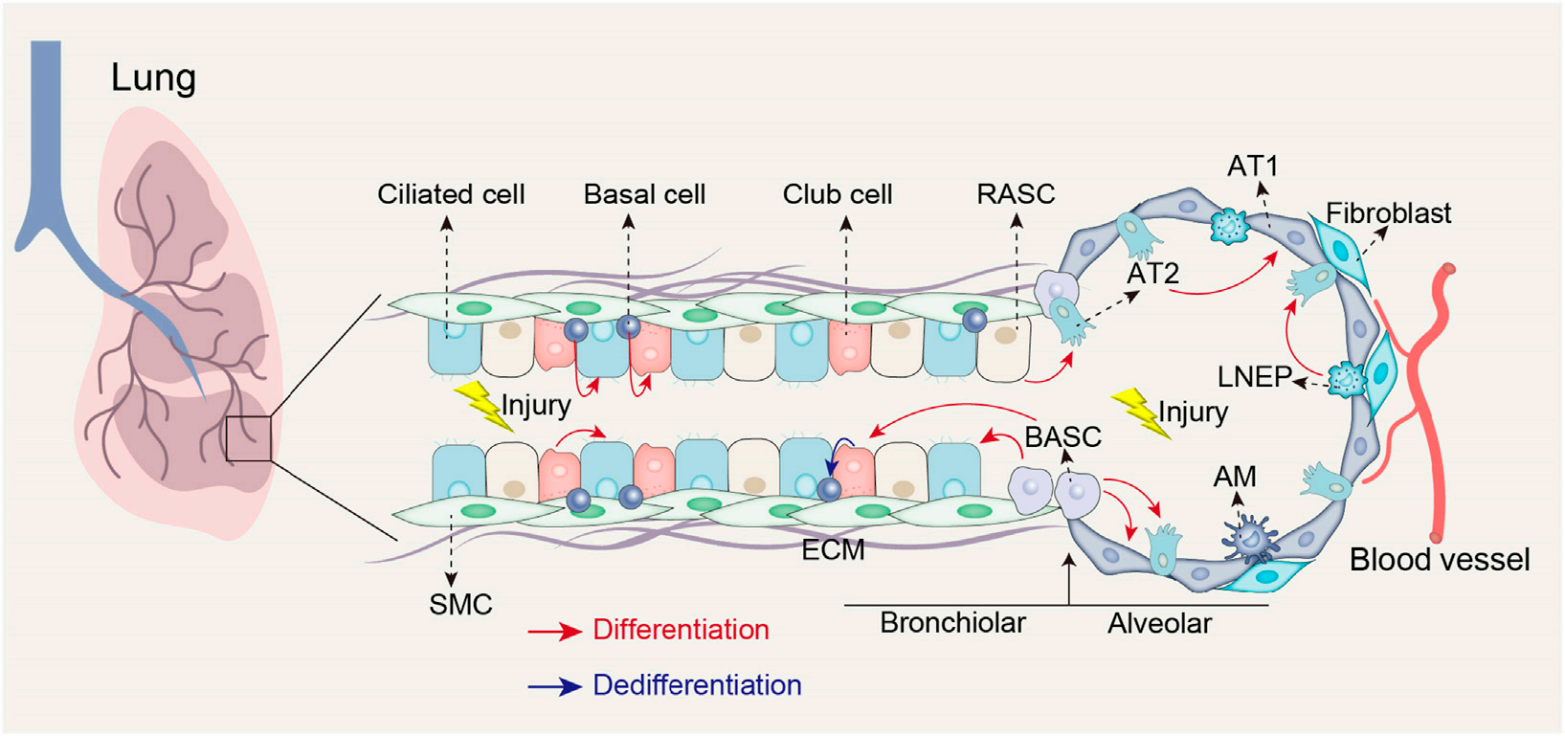
Stem cell populations involved in lung repair and regeneration following injury. Following airway injury, basal cells can differentiate into club cells and ciliated cells; club cells can differentiate into ciliated cells and possess the ability to dedifferentiate into basal cells when basal cells are depleted; BASCs can differentiate into both club cells and ciliated cells. Following alveolar injury, BASCs contribute to the replenishment of both AT1 and AT2 cells; LNEPs have the potential to differentiate into AT2 cells; RASCs can differentiate into AT2 cells, while AT2 cells themselves can differentiate into AT1 cells. AT1, Type 1 alveolar epithelial cells; SMC, Smooth muscle cell; ECM, Extracellular matrix; AM, Alveolar macrophages.

Impaired lung regeneration and repair processes can contribute to the development of lung fibrosis after injury (Basil et al., 2020). The lungs function as the primary organs of the respiratory system, taking in over 10,000 L of air daily to facilitate oxygen uptake into the bloodstream and the elimination of carbon dioxide (Neupane et al., 2020; Qiu et al., 2023). As organs in direct contact with the external environment, the lungs are susceptible to injury from various factors such as environmental pollution, smoking, chemical substances, and viral and bacterial infections (Zhang et al.; Deng et al., 2023). Following injury, the lungs possess a regenerative capacity and initiate a repair program by mobilizing various type of stem cells, including type 2 alveolar epithelial (AT2) cells, basal cells, club cells, lineage-negative epithelial progenitors (LNEPs), bronchioalveolar stem cells (BASCs), and respiratory airway secretory cells (RASCs) (Figure 1) (Meng et al., 2023). Effective lung repair and regeneration are crucial biological processes for restoring the normal physiological function of lungs after injury. However, chronic damage or disease can impede the lung regenerative abilities, resulting in fibrosis and functional impairment (Basil et al., 2020). Understanding of the cellular and molecular mechanisms underlying lung regeneration and repair is essential for comprehending the pathogenesis of lung fibrosis and developing effective therapeutic strategies.

The cellular and molecular mechanisms of lung regeneration, repair, and fibrosis are currently not fully understood. This Research Topic includes eight original articles and one review article that enhance and expand our knowledge regarding theses mechanisms. The findings contribute to our understanding of lung regeneration, repair, fibrosis, and offer potential new prognosis and therapeutic approaches for lung fibrosis and associated disorders.

Lung fibrosis and chronic obstructive pulmonary disease (COPD) occur due to lung injury and inadequate repair processes (Basil et al., 2020). Lung regeneration and repair following injury depends on the interaction between epithelial progenitor cells and fibroblasts in the alveolar stem cell niche. However, distorted Wnt/β-catenin signaling in fibroblasts may disrupt the crucial interactions between epithelial cells and fibroblasts, leading to impaired lung regeneration and repair (El Agha and Thannickal, 2023). In this regard, Khedoe et al. investigated the impact of cigarette smoke (CS) on Wnt/β-catenin signaling in fibroblasts and its effect on lung epithelial organoid formation. They exposed fibroblasts to CS extract (CSE) and an endoplasmic reticulum (ER) stress inducer Thapsigargin (Tg). CSE induced oxidative stress, while Tg stimulated the integrated stress response (ISR) and the unfolded protein response. Both CSE and Tg suppressed Wnt/β-catenin signaling and impaired the ability of fibroblasts to support lung epithelial organoid formation. Moreover, treatment with the Wnt activator reversed this inhibitory effect. These findings demonstrated that CSE exposure induces oxidative stress and hampers lung epithelial organoid formation via inhibiting the Wnt/β-catenin signaling pathway in fibroblasts.

Moreover, airway organoids offer an ideal model for studying the cellular and molecular mechanisms underlying airway epithelium regeneration and repair (van der Vaart and Clevers, 2021). Gentemann et al. employed airway organoids combined with femtosecond laser-based nanosurgery to investigate airway repair at a high spatio-temporal resolution, which is challenging to achieve *in vivo*. They found that the repair of airway organoids after cell ablation involves crucial mechanisms regulating native airway epithelial wound healing. This *in vitro* airway injury model provides a new approach to studying airway repair following localized injury and offers valuable insights into the single-cell level mechanisms driving epithelial repair.

Early intervention plays a crucial role in effectively treating IPF. However, the prognosis for individuals diagnosed with IPF is currently discouraging. To improve outcomes, it is essential to identify precise biomarkers, especially during the early stages of the disease, to enable timely therapeutic interventions (Moss et al., 2022). Zhang et al. integrated expression datasets from bulk tissue and single-cell datasets to analyze gene expression patterns in IPF. They found differentially expressed switch genes that showed correlations with clinical indicators, with the midkine (MDK) gene emerging as a particularly strong marker for the disease. The cellular communication-related genes of MDK were found to be differentially expressed in epithelial cells. A midkine score was calculated using MDK and its related genes, and machine learning models were developed to predict IPF using bulk gene expression datasets. The midkine score showed correlations with clinical indexes, and the machine learning model achieved a high level of accuracy in classifying IPF. These findings not only provide a new biomarker for IPF diagnosis but also offer valuable insights into the pathogenesis of the disease.

Besides MDK, cuproptosis-related genes (CRGs) may also serve as biomarkers for diagnosing IPF. Metal ions, including iron and copper, play crucial roles in cell metabolism, survival, and death. Alongside iron-dependent ferroptosis, cuproptosis has emerged as a recently identified form of programmed cell death. Cuproptosis is characterized by its dependence on copper, the accumulation of fatty acylated proteins, and the reduction of iron-sulfur cluster proteins (Stockwell, 2022; Tang et al., 2022; Xu et al., 2023). Shi et al. investigated the association between cuproptosis and IPF. They identified a positive correlation between activated dendritic cells and CRGs such as lipoyltransferase 1 (*LIPT1*), lipoic acid synthetase (*LIAS*), glutaminase (*GLS*), and dihydrolipoamide branched chain transacylase E2 (*DBT*). They also found correlations between CRGs and immune cell infiltration, emphasizing the significance of immune heterogeneity in IPF patients with distinct cuproptosis clusters. Moreover, an eXtreme Gradient Boosting (XGB) machine-learning model was developed for IPF diagnosis, exhibiting promising performance with lower residuals, a higher area under the curve, and validation using external datasets. These findings offer novel insights into the relationship between cuproptosis and IPF, as well as a potential diagnostic model for IPF patients.

In addition to CRGs, chemokines hold potential as biomarkers for diagnosing IPF. Chemokines, which are small proteins secreted by various cell types, play a crucial role in lung repair and fibrosis (Liu et al., 2021). Zhao et al. investigated the potential of a chemokine-related gene signature as a biomarker for IPF diagnosis. They successfully identified eleven chemokine-related genes that effectively differentiated IPF patients from controls. Additionally, two IPF subtypes were identified based on chemokine-related gene expression, with subtype 1 exhibiting higher pulmonary function parameters and stromal scores compared to subtype 2. Notably, altered expression of chemokine-related genes was observed in both bleomycin-induced mice and transforming growth factor beta-1 (TGFβ-1)-induced fibroblast cells. These findings suggest the potential of the identified chemokine-related genes as biomarkers for IPF and shed light on their involvement in the disease’s pathogenesis.

IPF and scleroderma-associated interstitial lung disease (SSc-ILD) are chronic fibrotic diseases that share common fibrosis pathways. SSc-ILD occurs in individuals with scleroderma, an autoimmune disease, and affects the lungs (Fields et al., 2023). It is characterized by inflammation and fibrosis in the lungs, resulting in progressive scarring and impaired lung function. The current treatment options for SSc-ILD involve non-specific immunosuppressive drugs and anti-fibrotic agents. However, these therapies have varying effectiveness, high costs, and potential side effects (Vonk et al., 2021). Woo et al. discussed the potential of targeting the nucleotide-binding domain and leucine-rich repeat protein-3 (NLRP3) inflammasome signaling pathway and the associated cytokines, including tumour necrosis factor α (TNFα), interleukin-1β (IL-1β), and interleukin-18 (IL-18), as novel therapeutic approaches for SSc-ILD. This review highlights that modulating these factors could offer new strategies for treating SSc-ILD.

SARS-CoV-2 infection, the virus responsible for COVID-19, can also contribute to lung injury and fibrosis. Individuals with diabetes and hypertension have been found to be susceptible to lung injury (Fuso et al., 2019; Rajasurya et al., 2020). Zhang et al. found that fibroblasts are upregulated in individuals with diabetes, hypertension, and hypertension-diabetes comorbidity who were infected with SARS-CoV-2, leading to reduced lung function. They identified specific genes associated with enhanced endothelial cell activation in fibroblasts and suggested kringle containing transmembrane protein 1 (*KREMEN1*) as a potential receptor for the activation of fibroblasts. Comparing Pirfenidone and Nintedanib, they propose Nintedanib as a more suitable treatment for COVID-19 patients with diabetes and hypertension and fibrotic lung lesions. These findings provide new insights into the modulation of fibroblasts during SARS-CoV-2 infection in comorbid conditions.

In addition to lung fibrosis and COPD, asthma, a prevalent chronic lung disease characterized by airway hyperresponsiveness and persistent airway inflammation, can also lead to reduced lung function. Globally, approximately 300 million people are affected by asthma. While medication is the primary approach for managing asthma, physical therapy, which includes exercise therapy, respiratory exercises, and muscle training, has proven to be effective (Brusselle and Koppelman, 2022). Zhou et al. has revealed that regular exercise can improve lung function, immune responses, and quality of life in children with asthma. Swimming and aerobic exercise have been identified as particularly beneficial for children with asthma. It is recommended to engage in these activities at least 2 to 3 times per week for a duration of 8 weeks, with each session lasting 40–60 min.

BALB/c and C57BL/6 mouse strains are commonly utilized in respiratory disease research, including studies on asthma. Among these strain, BALB/c mice have been observe to exhibit higher airway responsiveness than C57BL/6 mice (Duguet et al., 2000). Zeng et al. investigated the underlying mechanism using precision-cut lung slices. They found that BALB/c mice exhibited stronger small airway contraction and faster Ca^2+^ oscillations in airway smooth muscle (ASM) cells when exposed to agonists. This was attributed to increased store-operated Ca^2+^ entry (SOCE), resulting from elevated expression of SOCE components (STIM1, Orai1) in BALB/c mice’s small airway ASM cells. The mathematical model further supported that the elevated SOC current may lead to enhanced agonist-induced Ca^2+^ oscillations. These findings suggest that the inherently higher SOC activity in BALB/c mice contributes to the enhanced frequency of Ca^2+^ oscillations in ASM cells, stronger small airway contraction, leading to grater airway responsiveness compared to C57BL/6 mice.

In summary, the publications within this Research Topic have the potential to improve our understanding of the cellular and molecular mechanisms involved in lung regeneration, repair, and fibrosis. Furthermore, the papers make valuable contributions towards advancing the treatment options available for lung fibrosis and improving the diagnosis of individuals afflicted by this disease.
